# Profound Intraoperative Metabolic Acidosis and Hypotension in a Child Undergoing Multilevel Spinal Fusion

**DOI:** 10.1155/2009/190263

**Published:** 2009-10-18

**Authors:** Mohanad Shukry, Jonathan A. D'Angelo, Minal Joshi, Jorge A. Cure, Alberto J. de Armendi

**Affiliations:** Department of Anesthesiology, The Children's Hospital, University of Oklahoma Health Sciences Center, 750 North East 13th Street, Suite 200, Oklahoma City, OK 73104, USA

## Abstract

The prone position may cause cardiovascular system depression. Yet, the mechanisms involved and preemptive measures are not well understood (Edgcombe et al. (2008)). During spinal surgery in the prone position, hypotension may occur. Implicated factors include prolonged abdominal compression impeding venous return resulting in increased blood loss, decreased cardiovascular reserve, and the use of total intravenous anesthesia (TIVA) which has been shown to blunt the sympathetic response more than inhalation anesthesia. We present a case of hypotension during spinal surgery with all its challenges. Hypotension and acidosis persisted despite all supporting measures, and only to improve with supine positioning. Differential diagnosis for such an event are discussed. Although abdominal compression may not be obvious before the start of surgery, compressing the spine during surgery may lead to abdominal compression and hypoperfusion to abdominal organs.

## 1. Introduction

The prone position may cause cardiovascular system depression. Yet, the mechanisms involved and preemptive measures are not well understood [1]. We present a case of hypotension during spinal surgery with all its challenges.

## 2. Case Report

A 36 kg, 12-year-old boy with a past medical history significant for cerebral palsy, epilepsy, hydrocephalus, cortical blindness, and neuromuscular atrophy presented for posterior spinal fusion. Past surgical history included multiple ventricular peritoneal shunts, gastric fundoplication, and bilateral hip surgeries. The patient was receiving divalproex sodium for seizures. The physical exam was unremarkable.

**Figure 1:**
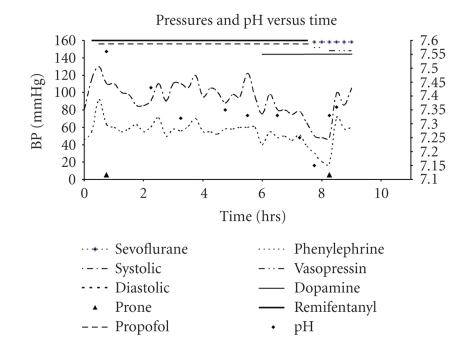
Vital signs and medications during surgery.

Anesthesia was induced with sevoflurane in a mixture of nitrous oxide and oxygen. An uncomplicated tracheal intubation was performed followed by placement of peripheral intravenous access, central line, and arterial line. Following prone positioning and avoiding abdominal compression, total intravenous anesthetic (TIVA) was administered with propofol 180 mcg kg^−1^min^−1^ and remifentanil 2 mcg kg^−1^min^−1^. As combined motor and somatosnesory evoked potential (SSEP) monitoring were used, TIVA with avoiding muscle relaxants was the anesthetic plan of choice. Two hours later, hypotension ensued with mean arterial pressure (MAP) of 46–50 mmHg. Propofol and remifentanil were decreased to 150 mcg kg^−1^min^−1^ and 1 mcg kg^−1^min^−1^, respectively. Phenylephrine 50 mcg and calcium gluconate 100 mg IV were administered. A unit of packed blood cells (PRBC) was administered. Over the following 6 hours, dopamine infusion (5 mcg kg^−1^min^−1^) was initiated; propofol and remifentanil were decreased to 75 mcg kg^−1^min^−1^ and 0.075 mcg kg^−1 ^min^−1^, respectively. Phenylephrine 7000 mcg, calcium gluconate 1200 mg, sodium bicarbonate 100 mEq and calcium chloride 600 mg, 5200 mL normal saline, 3 units PRBCs, and 840 mL from cell saver were administered. Two hours later, the patient became hemodynamically unstable again with blood pressures of 48–65/18–38 mmHg and HR 122–135 beat min^−1^ along with worsening metabolic acidosis. Propofol infusion was stopped, dopamine infusion was increased to 7 mcg kg^−1^min^−1^ and a phenylephrine infusion was initiated at 5 mcg kg^−1^min^−1^. Bicarbonate 100 mEq and hydrocortisone 100 mg were administered. TIVA was stopped and sevoflurane (1%) was initiated. Phenylephrine was stopped and vasopressin infusion was initiated along with one more unit of PRBCs. When the patient was turned into supine, his BP stabilized to 100/72 mmHg within minutes (Figure 1), he remained stable and was transferred to the pediatric intensive care unit with his trachea intubated. The patient was weaned off the vasopressors overnight, and his trachea was extubated in the following morning without sequelae.

## 3. Discussion

A 12-year-old child with cerebral palsy and severe neuromuscular scoliosis who underwent posterior spinal fusion and instrumentation developed severe hypotension and metabolic acidosis that was refractory to crystalloid and vasopressors until he was repositioned from prone to supine. Hypotension may occur during spinal surgery in the prone position; implicated factors include prolonged abdominal compression impeding venous return resulting in increased blood loss, decreased cardiovascular reserve, and the use of total intravenous anesthesia (TIVA). The differential diagnosis for persistent hypotension and progressively worsening metabolic acidosis was considered in the following order: (1) hypovolemia, (2) increased abdominal pressure causing inferior vena caval compression and visceral ischemia, (3) propofol infusion syndrome, and (4) acute adrenal insufficiency.

Blood loss was estimated to be 2300 mL over the first 6.5 hours of surgery up until the hypotension occurred, possibly causing hypovolemic shock refractory to vasopressors as well as worsening acidosis. However, 5.2 L of normal saline, 4 units of PRBCs, and 840 mL cell saver blood were administered over the same period and that should have been adequate. Administering a high volume of normal saline, approximately 30 mL kg^−1^h^−1^, has been shown to cause significant hyperchloremic metabolic acidosis [2]. However, our patient only received 16 mL kg^−1 ^h^−1^, decreasing the likelihood of hypercholremic acidosis. In retrospect, serum chloride electrolyte should have been measured during the surgery. Additionally, initial high doses of propofol and remifentanyl (180 mcg kg^−1^min^−1^ and 2 mcg kg^−1^min^−1^ consecutively) would have lead to such initial decrease in BP in a mildly hypovolemic patient due to their vasodilation properties [3].

Prone position causes decreased cardiac index due to a decrease in stroke volume. This decrease in stroke volume has been attributed to various causes: increased intra-thoracic pressure causing a decrease in left ventricular compliance [4], a decrease in arterial filling leading to an increase in sympathetic activity via the baroreceptor reflex [1], and inferior vena cava obstruction. Mechanical compression on the heart leading to hypotension has also been reported in patients with chest deformities such as pectus excavatum [5] which our patient did not have. In our patient, severe hypotension occurred minutes following spinal instrumentation and rod placement, in which intense pressure and force was exerted to the posterior spinal region (T2-L2). Obstruction of the inferior vena cava is a well recognized complication of prone positioning and is exacerbated by any degree of abdominal compression, leading to decreased cardiac output and increased bleeding, venous stasis, and consequent thrombotic complications [1]. These effects have also been implicated in precipitating hepatic ischemia with progressive metabolic acidosis and elevated liver enzymes after prolonged surgery in the prone position [6] with subsequent resolution. Although the patient's abdomen was freely hanging without any pressure, this could not be true during surgery and forceful compression on a malleable spine.

Propofol infusion syndrome (PIS) can cause severe metabolic acidosis, rhabdomyolysis, cardiac failure, and renal failure [7]. Recently, there have been an increasing number of case studies implicating propofol as the cause of reversible mild acidosis in noncritically ill patients. We were administering a moderately high dose of propofol, averaging 8.9 mg kg^−1 ^h^−1^, until we switched to sevoflurane. Serial ABGs (see Table 1), allowed us to monitor a consistent and progressive drop in pH, HCO_3_, and BE over the course of the surgery and even a more acute drop during the time of hemodynamic instability. Postoperative CPK was not obtained to confirm rhabdomylosis, but it is not unusual for CPK to be elevated following spine surgeries without rhabdomylosis. 

Lastly, we also considered acute adrenal insufficiency as a contributing cause of the unforeseen problems that occurred. Acute adrenal insufficiency presents with acidosis, hypotension, and an adequate urine output. However hyponatremia, hyperkalemia, and hypoglycemia, are other symptoms that did not develop in our patient. Nonetheless, hydrocortisone 100 mg was administered shortly following the severe hypotensive episode.

## 4. Conclusion

Children undergoing spinal procedures can develop hemodynamic instability due to multiple factors such as hypovolemia, prone position leading to vena cava compression and metabolic acidosis. Compression of inferior vena cava during the procedure could take place at any point due to pressure effects from manipulation of the spine. We believe that this was the most plausible cause in our patient.

## Figures and Tables

**Table 1:** Laboratory values over time during surgery. Induction time is zero time.

Time (hrous)	0.75	2.25	3.25	4.75	5.5	6.5	7.25	7.75	8.25	8.5
PH	7.56	7.43	7.32	7.35	7.33	7.33	7.25	7.15	7.33	7.36
PaCO_2_ (torr)	27	31.9	37.9	29.3	31.5	32.5	36.6	38.7	35.7	40.2
PaO_2_ (torr)	466	285	260	274	269	296	182	125	134	159
BE (mEq/L)	2	−3	6	−9	−9	−9	−11	−15	−7	−3
HCO_3_ (mEq/L)	24	21.3	19.7	16.3	16.6	16.9	15.9	13.4	18.6	22.6
SaO_2_ (%)	100	100	100	100	100	100	99	98	99	99
Na^+^ (mEq/L)	136	140	145	145	145	148	149	147	152	152
K^+^ (mEq/L)	3.7	3.4	3.3	3.8	3.9	4.1	4	4.7	4.6	4.3
Ionized Ca^+2^ (mmol/L)	1.1	1.16	1.15	1.15	1.16	1.19	1.19	2.14	1.18	1.09
Glu (mg/dl)	99	100	98	112	112	160	151	200	183	222
Hct (%)	35	28	25	26	33	32	29	44	41	42
Hgb (g/dl)	11.9	9.5	8.5	8.8	11.2	10.9	9.9	15	13.9	14.3
